# A Case of Synchronous Multiple Primary Malignancies Involving Multiple Myeloma and Pharyngeal Plasmacytoma in an Elderly Male: A Case Report and Review of the Literature

**DOI:** 10.7759/cureus.33358

**Published:** 2023-01-04

**Authors:** Muhannad Q Alqirnas, Abdulmajeed M Alqahtani, Yazeed H Alotaibi, Moustafa S Alhamadh, Rakan B Alanazi, Abdulrahman D Alharbi, Fawaz A Alhamied, Ohoud Z Aljarbou, Mohammad Alkaiyat, Fouad Sabatin, Abdullah Mohammed Altwim

**Affiliations:** 1 College of Medicine, King Saud Bin Abdulaziz University for Health Sciences, Riyadh, SAU; 2 Pathology and Laboratory Medicine, Ministry of the National Guard Health Affairs, King Abdulaziz Medical City, King Abdullah International Medical Research Center, Riyadh, SAU; 3 Medical Oncology, Ministry of the National Guard Health Affairs, King Abdulaziz Medical City, King Abdullah International Medical Research Center, Riyadh, SAU

**Keywords:** pharyngeal tumor, multiple myeloma, solitary extramedullary plasmacytoma, saudi arabia, pharyngeal plasmacytoma

## Abstract

Monoclonal plasma cells form the solitary neoplasm known as solitary plasmacytoma. Isolated extramedullary plasmacytoma is less common than solitary bone plasmacytoma. An elderly male presented with coughing blood and was diagnosed with pharyngeal plasmacytoma with synchronous multiple myeloma. Herein, we present this challengingly rare case to increase awareness of this unusual entity.

## Introduction

Monoclonal plasma cells form the solitary neoplasm known as solitary plasmacytoma (SP) [1-3]. It comes in either a bony or an extramedullary form. Extramedullary plasmacytoma (EMP) makes up approximately 4% of all plasma cell tumors, usually developing in the nasopharynx, larynx, or sinuses, but occasionally sporadically developing in the skin, lymph nodes, gastrointestinal tract, and soft tissues [4]. EMP is less prevalent than bony plasmacytoma, which has a larger incidence. Schridde was the first to describe EMP, an uncommon tumor, in 1905 [5]. It has been estimated that the prevalence of SP is 0.15 per 100,000 people, making it a rare condition [6]. SP is diagnosed with tissue biopsy, which is the most reliable method [7]. Its biopsy sample will most likely reveal a characteristic thick plasma cell with a monotonous appearance [8]. Additionally, both diseases also differ in prognosis although both forms respond well to treatment. The prognosis for solitary bony plasmacytoma is worse than that for solitary EMP due to its higher rate of development to multiple myeloma (MM) [9]. The diagnosis of EMP, a plasma cell malignancy characterized by a soft tissue mass of monoclonal plasma cells, is made after other systemic plasma cell disorders have been ruled out [5].

Here, we present a case of a solitary EMP that affected the pharynx and resulted in coughing blood as the initial clinical symptom, followed by chest discomfort. To the best of our knowledge, this is the first case report of EMP in the pharynx with synchronous MM. To better understand the clinical signs and symptoms of EMP affecting the pharynx, we also analyzed prior studies. The patient gave written, explicit permission for the publication of the case report and any supplemental pictures. Herein, we present this challengingly rare case to increase awareness of this unusual entity.

## Case presentation

A 63-year-old male non-smoker, a known case of hypertension, type 2 diabetes mellitus, and coronary artery disease post percutaneous coronary intervention four months ago presented to our emergency department complaining of coughing blood. He was in his usual health until three hours ago and started coughing blood proceeded by chest discomfort. History was negative for previous similar attacks, bleeding disorders, stool and urine color changes, abdominal pain, pain with swallowing, nasal obstruction, and any change of level of consciousness, and was negative for smoking. The patient's past medical history was remarkable for medically treated kidney stones six months ago, and his family history was noncontributory. He was on aspirin post cardiac catheterization, with no history of nasal or oral surgeries.

On examination, he was conscious, alert, and oriented to time, place, and person, and he did not exhibit any signs of respiratory distress. He had an elevated blood pressure of 171/81 mmHg, a respiratory rate of 25, an oxygen saturation of 96% on room air, and a temperature of 37.9°C. His cardiopulmonary, neurological, abdominal, and genitourinary examinations were normal. However, an ENT examination revealed active postnasal bleeding with a mass hanging from the nasopharynx. He was taken to the operating room immediately for control of bleeding, better inspection, and mass removal under general anesthesia. After the operation theater, there was no more oral bleeding and the patient was vitally stable, and he was admitted for further management.

Laboratory investigations displayed leukocytosis (12 x 109), but normal levels of hemoglobin (171 gm/l), with platelets at 234 x 109/L, hyponatremia at 130 mmol/L, and critical random glucose level of 27.9 mmol/L. The rest of the results can be found in Table 1. Bence Jones protein, with serum plasmapheresis and immunofixation, was absent with normal plasmapheresis.

**Table 1:** Results of the laboratory investigation upon the patient's admission WBC = white blood cells; HGB = hemoglobin; ESR = erythrocyte sedimentation rate; LDH = lactate dehydrogenase; BUN = blood urea nitrogen; CO2 = carbon dioxide; eGFR = estimated glomerular filtration rate.

Lab name	Value
WBC	12 x 109
HGB	171 gm/l
ESR	38 mm/hr
LDH	213 U/L
Platelets	234
Natrium	130 mmol/L
Random glucose	27.9 mmol/L
Creatinine	240 umol/L
BUN	18.4 mmol/L
Uric acid	479 umol/L
CO2	19
Anion gap	17
eGFR	25
Calcium	2.14 mmol/L
Adjusted calcium	2.12 mmol/L
Plasma cell	2%
Beta-2 microglobulin	5.04 g/L
IgG	11 g/L
IgA	4.61 g/L
IgM	0.29 g/L

**Figure 1:**
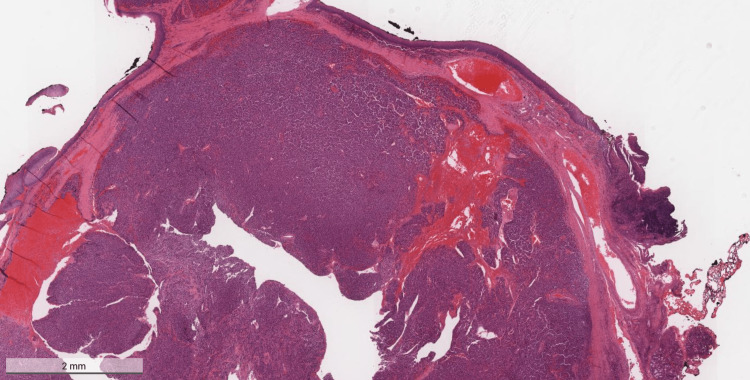
Hematoxylin & eosin (H&E) stained sample (magnification x2) of the mass that was excised from the pharynx The figure shows diffuse sheets of atypical, mostly monotonous, plasma cells with occasional binucleate forms. Only occasional mitoses are seen.

**Figure 2:**
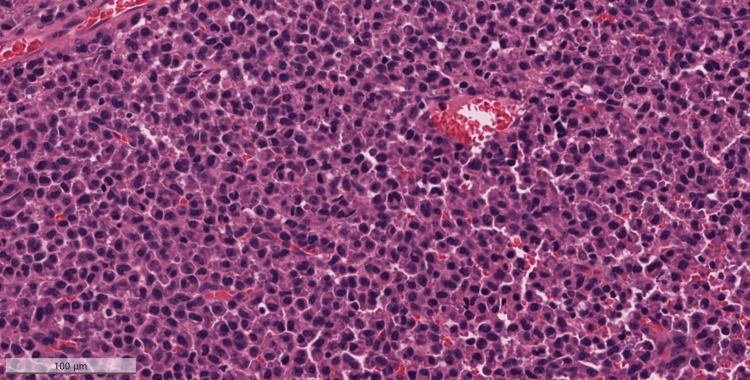
Hematoxylin & eosin (H&E) stained sample (magnification x20) of the mass that was excised from the pharynx The figure shows diffuse sheets of atypical, mostly monotonous, plasma cells with occasional binucleate forms. Only occasional mitoses are seen.

Upon admission, the surgical tissue biopsy from the pharyngeal mass showed diffuse sheets consisting of plasmacytoma plus multiple myeloma deposits (Figures 1, 2). The neoplasm was formed of diffuse sheets of atypical, mostly monotonous, plasma cells with occasional binucleated forms and no necrosis.

**Figure 3:**
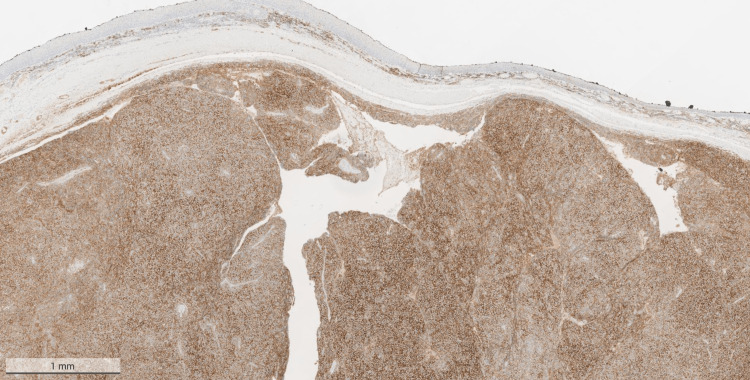
Positive cells on CD38 stain of the mass that was excised from the pharynx

**Figure 4:**
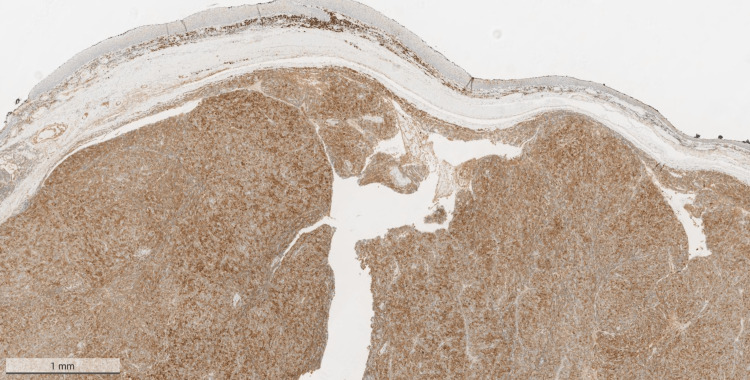
Positive CD79a stain on the mass that was excised from the pharynx

**Figure 5:**
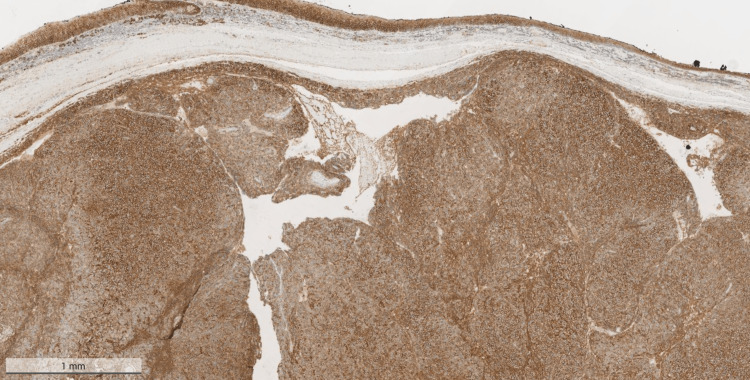
Positive CD138 stain on the mass that was excised from the pharynx

**Figure 6:**
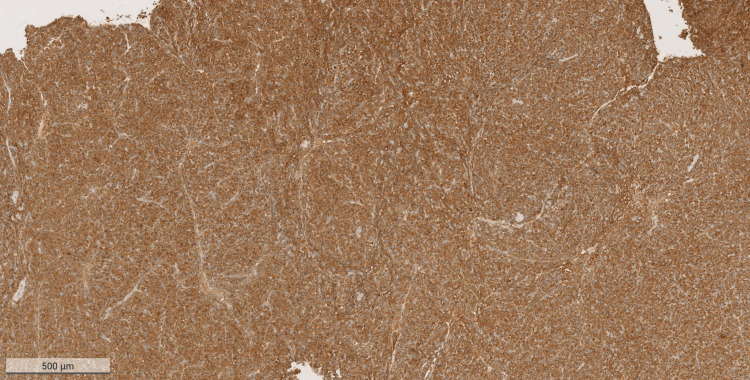
Image showing some restriction on the light chain of the mass that was excised from the pharynx

**Figure 7:**
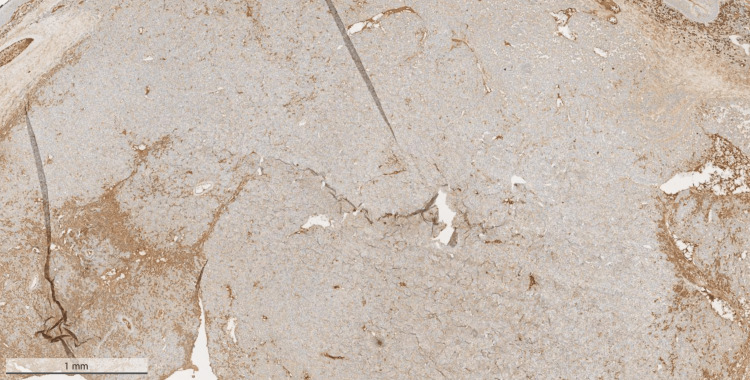
No staining on kappa of the mass that was excised from the pharynx was present

On immunohistochemistry, the lesional cells were positive for CD38 (Figure 3), CD79a (Figure 4), and CD138 (Figure 5), and were showing lambda light chain restriction (Figure 6). However, there was no staining on Kappa (Figure 7).

Bone marrow aspiration was obtained and had no evidence of plasma cell dyscrasias and cytogenic/fluorescence in situ hybridization (FISH) results were negative.

**Figure 8:**
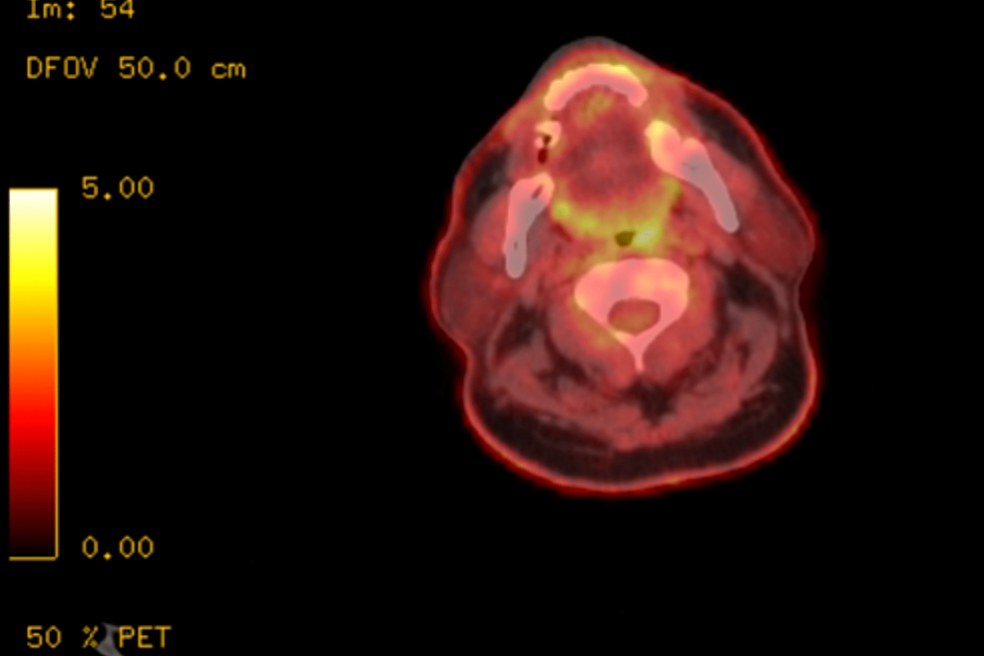
Positron emission tomography (PET) scan of the oropharynx showing increased uptake in the left oropharynx

After mass removal, the whole body positron emission tomography (PET) scan was notable for mildly increased uptake in the left oropharynx (Figure 8), which was nonspecific but residual tumor could not be excluded. It also showed two small right level II nodes, with mild diffuse bone marrow activity throughout the skeleton including a tiny lucency in the L1, and a right-sided hydronephrosis with renal stones was also appreciated.

The patient was diagnosed with an isolated solitary plasmacytoma of the pharynx. He was scheduled for local radiotherapy to the oropharynx. He received 25 sessions of radiotherapy without complication.

## Discussion

A rare subset of plasma cell dyscrasias includes solitary EMP, i.e., in soft tissues and solitary bony plasmacytoma [4]. According to an examination of data from the Anderson Cancer Center between 1963 and 1996, 1272 (94%) patients had MM, 60 (4%) had SP, and 22 (2%) had EMP [10]. As solitary EMP of the pharynx is rare so it has been challenging to compile sizable cohorts and conduct clinical trials that might alter the SP therapy paradigm. SP is characterized by a single mass of clonal plasma cells, no or little bone marrow plasmacytosis, and no additional symptoms except those resulting from the original lesion [11]. Solitary EMP can be linked to MM [12,13]. Twenty-five years ago, Wiltshaw noted a connection between MM and EMP, as well as a propensity for submucosal extension, the frequency of dissemination to other soft tissues, and the uncommon affliction of bone marrow [14]. Our patient also has synchronous MM.

The location of the tumor has a significant impact on the clinical presentation of SP. Solitary EMP patients typically present to the clinic with symptoms of pain, trouble swallowing, shortness of breath, epistaxis, and nasal congestion since the affected areas are frequently the nasopharynx, sinuses, and larynx [12]. In this instance, SP presented with the symptom of coughing up blood proceeded by chest pain and the location was the pharynx. There was no such case in the literature, as far as was known. A 3:1 male-to-female ratio and a median age of 55 years are associated with EMP presentations [5]. Our patient was also a 63-year-old male. ENT examination indicated postnasal bleeding that was active and there was a mass hanging from the nasopharynx. Mass was removed under general anesthesia and his bleeding was controlled. Tissue biopsy is the definitive method of diagnosis [15]. In our patient, tissue biopsy from the pharyngeal mass showed diffuse sheets consisting of plasmacytoma plus MM deposits.

According to an analysis in a study, the first two years following diagnosis carry the highest probability of conversion to MM; however, conversion has also been documented more than 15 years later [16]. Three patients acquired MM in the first two years, and one subject did so 12 years later, according to the literature. Although there is disagreement over the high-risk factors for conversion to MM, patients who have already undergone conversion have a dismal prognosis; less than 10% of patients survive for 10 years [4]. Therefore, the development of MM may be a factor in bad prognosis or a factor that determines survival. This might be because most cases only receive a short follow-up. As a result, regular MM screening and follow-up are crucial.

In EMP, CT and/or MRI are used to evaluate adherent lymph nodes and bone structures that may be impacted particularly in the nasal cavity and maxillary sinuses [17]. A neck CT scan was performed after the mass was removed from our patient and it revealed no abnormal enhancement or residual masses. On the whole-body PET scan, the left oropharynx exhibited a modest increase in uptake, which was nonspecific but could not rule out residual tumor [18].

Radiotherapy has typically been the primary line of treatment for solitary EMPs since EMPs are radiosensitive [19]. In the literature, single-modality radiotherapy was the most popular form of treatment for EMP, followed by surgery. Recently, it was postulated and demonstrated that surgically based treatment including surgical resection could provide better survival outcomes than radiotherapy alone [4]. Some studies, on the other hand, found no survival advantage for one treatment approach over another and even advised against extensive surgery for EMP [5]. Our patient underwent local radiotherapy to the oropharynx and received 25 sessions without complication. The most effective management strategy for EMP is still up for debate. However, it is well acknowledged that chemotherapy is not a first-line treatment choice and that adjuvant chemotherapy is typically employed in patients with widespread or recurrent disease [4].

There is no agreement on the prognostic variables and course of treatment due to the disease's rarity [19]. The function of chemotherapy and surgery is also still up for dispute. However, we must keep in mind that the bulk of studies only examines the effectiveness of conventional chemotherapy on a small number of patients, and very few examples involving the effectiveness of innovative medications are recorded. Only surgical resection of the mass has been used to treat this patient followed by radiotherapy.

## Conclusions

Pharyngeal plasmacytoma with synchronous multiple myeloma is rare, especially in an elderly patient. The presentation is most likely vague, and the diagnosis is challenging. The diagnosis is made by a histopathological examination.
